# Isokinetic angle-specific moments and ratios characterizing hamstring and quadriceps strength in anterior cruciate ligament deficient knees

**DOI:** 10.1038/s41598-017-06601-5

**Published:** 2017-08-04

**Authors:** Hongshi Huang, Jianqiao Guo, Jie Yang, Yanfang Jiang, Yuanyuan Yu, Steffen Müller, Gexue Ren, Yingfang Ao

**Affiliations:** 1 Institute of Sports Medicine, Peking University Third Hospital, Beijing, 100191 China; 2 School of Aerospace Engineering, Tsinghua University, Beijing, 100084 China; 3 Departments of Sports Medicine and Orthopedics, University of Potsdam, Potsdam, 14469 Germany

## Abstract

This study is intended to find more effective and robust clinical diagnostic indices to characterize muscle strength and coordination alternation following anterior cruciate ligament (ACL) rupture. To evaluate angle-specific moments and hamstring (H)/quadriceps (Q) ratios, 46 male subjects with unilateral chronic ACL-rupture performed isokinetic concentric (c), eccentric (e) quadriceps and hamstring muscle tests respectively at 60°/s. Normalized moments and H/Q ratios were calculated for peak moment (PM) and 30°, 40°, 50°, 60°, 70°, 80° knee flexion angles. Furthermore, we introduced single-to-arithmetic-mean (SAM) and single-to-root-mean-square (SRMS) muscle co-contraction ratios, calculating them for specific angles and different contraction repetitions. Normalized PM and 40° specific concentric quadriceps, concentric hamstring strength in the ACL-deficient knee were reduced significantly (*P* ≤ 0.05). Concentric angle-specific moments together with Qe/Qc ratios at 40° (*d* = 0.766 vs. *d* = 0.654) identify more obvious differences than peak values in ACL ruptured limbs. Furthermore, we found SRMS-QeQc deficits at 40° showed stronger effect than Qe/Qc ratios (*d* = 0.918 vs. *d* = 0.766), albeit other ratio differences remained basically the same effect size as the original H/Q ratios. All the newly defined SAM and SRMS indices could decrease variance. Overall, 40° knee moments and SAM/SRMS ratios might be new potential diagnosis indices for ACL rupture detection.

## Introduction

Muscle strength is important to the health of an individual, critical for optimal performance in sports and for preventing certain serious sport-related injuries. Quadriceps strength deficits may be ubiquitous after anterior cruciate ligament (ACL) rupture and reconstruction^1^. The quadriceps is important for lower limb control during dynamic activity and quadriceps weakness could alter movement strategies potentiating re-injury, which may be hazardous to the patient^2^.

Isokinetic dynamometry-based strength evaluations both in healthy individuals and ACL-deficient (ACLD) patients allow establishment of baseline scores and progress of different phases. Most studies of muscle strength related to the ACL injury have used the peak moment as main outcome measure^3–5^. Quadriceps peak moment represents the value during knee extension where the individual could produce the highest force^6^. ACLD patients have shown about 14–25% quadriceps^5, 7^ and hamstring strength^7, 8^ deficits on the injured limb. However, the peak moment may give limited information about the muscle performance throughout the range of motion (ROM)^9^. The efficiency of muscle strength testing could be improved if the same angle could be used^10^. Especially, quadriceps contraction would produce a larger anterior tibial shear force when the knee flexion angle is less than 40°. Compared to the commonly used peak moment values, angle of less than 40° specific thigh muscle moment values provided more information on the strength deficits after ACL injury^11^. And Eitzen *et al*. revealed that the largest quadriceps deficits could be found at less than 40° knee angle for both potential copers and non-copers^5^.

The assessment of strength and strength balance about the knee has been used as an objective marker after ACL injury, reconstruction and subsequent rehabilitation. Therefore, a hamstring/quadriceps (H/Q) strength ratio based on peak moment values during a maximal voluntary contraction has traditionally been used to describe the potential for knee joint stabilization^12, 13^. The Hamstring concentric/Quadriceps concentric (Hc/Qc) ratios and the Hamstring eccentric/Quadriceps eccentric (He/Qe) ratios of peak moments have been studied in ACLD subjects^14^. Kannus reported that a greater difference of the Hc/Qc ratio between the ACLD and the sound limb was associated with a less successful outcome of rehabilitation^15^. However, the Hc/Qc ratio is not physiologic, because there is a wide discrepancy between the time and angle of the hamstring peak force and those of quadriceps. The peak moments generated by the knee extensors and flexors occur at about 60° and 20° of knee flexion, respectively^16^. In contrast to the Hc/Qc, the functional He/Qc ratio is similar to the kicking action, and the increase in the ACLD side may help the patients accomplish low intensity knee extension motion^17^.

Up to now, there is still few work related to the angle specific H/Q ratios, especially for ACLD patients. Aagaard *et al*. put forward the concept of “functional H/Q ratio”, and reported 30°, 40°, and 50° He/Qc ratio values^12, 13^. Coombs *et al*. have shown angle specific characteristics of He/Qc and Hc/Qc ratios throughout 90° range of motion^14^, but they did not present the optimal knee angle for analysis. Hiemstra *et al*. stated that the largest Hc/Qc ratio variations could be found at around 40° knee angle between normal and ACL reconstructed subjects^18^, yet no concentration was paid on ACLD patients. According to our previous results, the Hc/Qc, He/Qc and Qe/Qc ratios significantly changed on the ACLD side at 30° of knee flexion^17^. However, the optimal joint angle of the H/Q ratio which demonstrates the most obvious disparities between the healthy and ACLD limb needs to be clarified.

Moreover, muscle co-contraction should also be taken into consideration for ACL injury evaluation, due to that antagonist muscles must contract simultaneously to increase joint stability and protect ligaments during motion^19^. Numerous forms of indices have been introduced for muscle co-activation assessment of different joints^20–23^, and they are compared by Souissi *et al*. recently^24^. All of the indices have been based on muscle electromyography activities (EMG) or moments calculated by musculoskeletal models, and as has been pointed out, measurement of muscle co-contraction level based on joint moments might provide more reliable description of muscle actions^24^. All forms of the H/Q ratio directly compare the strength of two isolated muscle groups, however, they lack proper measurement on the simultaneous contraction strength of agonist and antagonist muscles. So new indices have been introduced in this paper based on quadriceps and hamstring muscle strength from isokinetic tests. They were computed by following the protocol of co-contraction index. Furthermore, nearly all the indices included linear combinations of muscle moments or EMG data, but nonlinear polynomial forms of cost functions have been more frequently used for solving the so-called muscle distribution problem due to the musculoskeletal system redundancy^25^. Therefore, the root-mean-square (RMS) form of quadriceps and hamstring muscle strength has been finally proposed to describe knee muscle coordination.

At 30° of knee flexion, which was important for knee dynamic function, there was a significant reduction in the moment of quadriceps at concentric and eccentric 60°/s produced by the deficient-side compared to the intact side^17^. According to our clinical experience, the H/Q ratio may demonstrate extremely high values when the quadriceps strength is close to zero. In addition, Ayala *et al*. determined the absolute reliability of conventional and functional H/Q ratios, and found that angle-specific H/Q ratios showed poor absolute reliability^26^. On the contrary, Hirai *et al*. have shown that the variance of muscle synergy indices of upper limbs was sufficiently small for each subject^27^. Therefore, it seems that new forms of robust hamstring and quadriceps muscle strength indices for decreasing standard deviation can be introduced based on the polynomial combination of hamstring and quadriceps muscle strength.

The purpose of this study was to determine the moments and ratios of the hamstring and quadriceps strength at 30°, 40°, 50°, 60°, 70°, 80° of knee flexion in 60°/s isokinetic contractions, trying to examine the differences of angle specific hamstring and quadriceps moments and muscle strength parameters between the healthy and ACLD limb, and find more robust clinical diagnosis indices to detect altered muscle coordination strategies with lower variance. We hypothesized that 30° and 40° H/Q ratios would show more effective statistical significance than peak moment H/Q values. In addition, we also hypothesized that the newly introduced angle specific muscle co-contraction indices (shown below) would reduce diversity and increase robustness than H/Q ratios.

## Methods

### Ethics statement and Subjects

The study protocol was approved by the Institutional Research Board of Peking University Third Hospital (IRB00006761–2013070) and the written informed consents were obtained from all subjects. This study was performed in 46 male subjects (30.1 ± 6.6 years, BMI 26.1 ± 4.0 kg/m^2^) with unilateral and symptomatic ACL deficiency (24 left, 22 right), who reported symptom of giving-way during pivoting or twisting activities while no symptom of pain, swelling or giving-way during straight line and level walking. Subjects were recruited to the study from the Institute of Sports Medicine, Peking University Third Hospital (Beijing, China) between June 2013 and December 2015. ACL deficiency was confirmed through physical examination by an orthopedic surgeon as well as through MRI examination. In addition, the status of each injured ACL was verified by an arthroscopic ACL reconstruction procedure. All patients had unilateral ACL rupture with minimal other tissue injuries that did not need surgical intervention. There was no history of injury, surgery or disease in the contralateral knees of the patients.

### Testing Procedure

Subjective evaluation included subjective International Knee Documentation Committee (IKDC)^28^, Lysholm and Tegner scores^29^ (IKDC 66.6 ± 15.1; Lysholm score 77.5 ± 15.6; Tegner score 4.1 ± 1.6). Those evaluations were single blinded and performed by the same experimenter. Objective evaluation included isokinetic quadriceps and hamstring muscle tests.

### Isokinetic muscle strength testing

Before each test, there was a 5-minute warmup cycling session (SCIFIT, American). Subjects were also asked to complete practice repetitions once or twice prior to each test series, separated by 90 seconds rest intervals. All isokinetic measurements were recorded by the same experimenter to avoid intertester variability. Isokinetic concentric and eccentric quadriceps and hamstring muscle tests in both the sound and ACLD knees were performed at 60°/s respectively, which were frequently referred to as a relevant and valid measurement for muscle performance in ACLD individuals^2, 30^. The reliability for isokinetic muscle testing of knee was previously reported to be adequate both for healthy subjects and for subjects with ACL deficiency^31, 32^.

**Figure 1:**
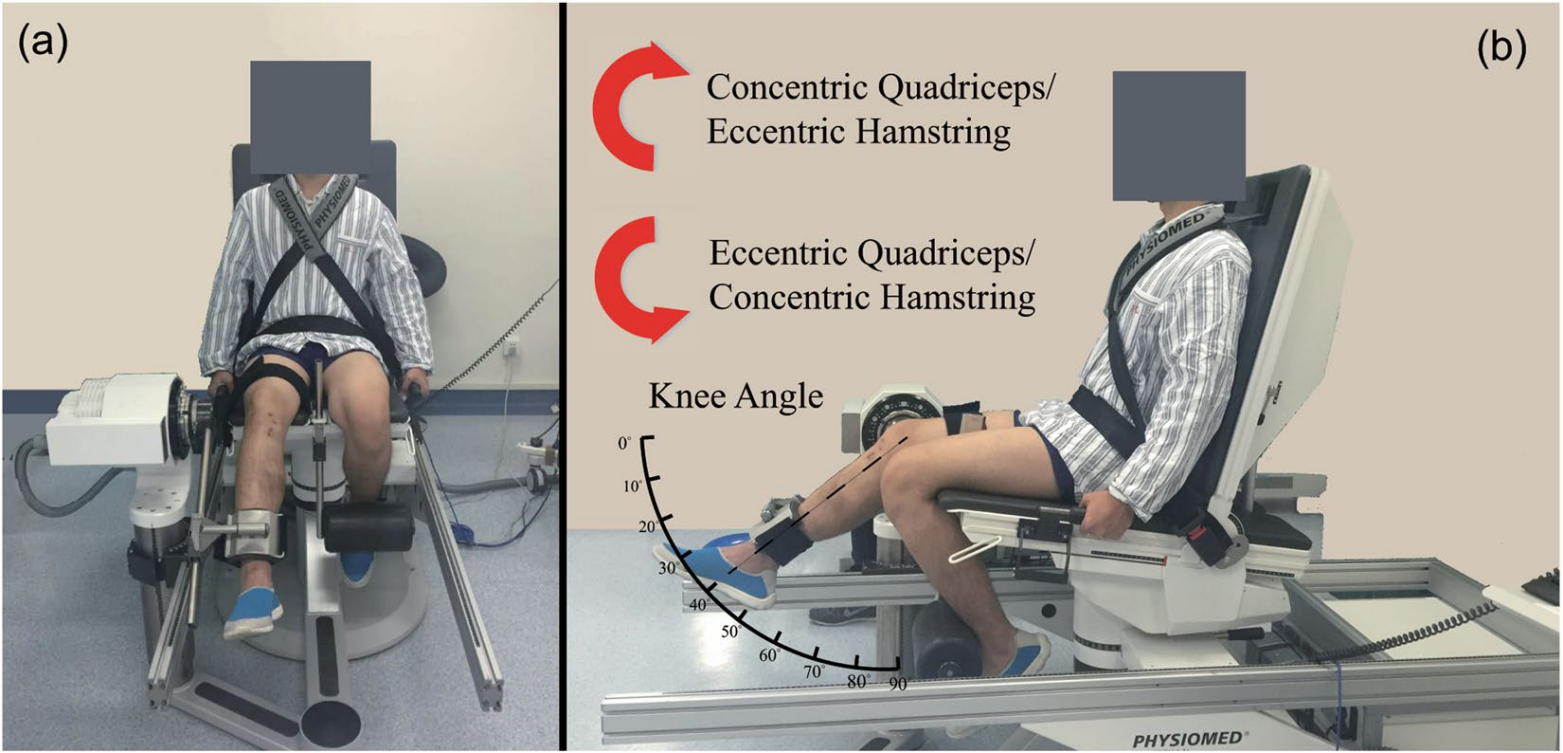
Illustrations of isokinetic test protocol. (**a**) An example of the isokinetic test by a subject and (**b**) an illustration of knee motion and the limb muscle contraction in the isokinetic test.

The thigh muscles of the uninvolved limbs were tested first by using an isokinetic dynamometer (Con-Trex MJ; Germany), as shown in Fig. 1. Subjects were comfortably seated on the dynamometer chair, with the hip joint at about 85° (0° = full extension). The distal shin pad of the dynamometer was attached proximal to the medial malleolus by using a strap. To minimize extraneous body movements during thigh muscle contractions, straps were applied across the chest, pelvis and mid-thigh. The alignment between the dynamometer rotational axis and the knee joint rotation axis (lateral femoral epicondyle) was checked at the beginning of each trial. Gravity effect moment was recorded on each subject throughout the range of motion and this was used to correct moment measurements during all tests. The participants were given standardized encouragement by the investigator, and were asked to position their hands on the bilateral handles on the side of the chair during the testing procedure. The ROM for the isokinetic trials was 70°, from 20° to 90° of knee flexion (0°corresponding to knee fully extended). Knee flexor and extensor trials were performed as discrete movements in both directions at a velocity of 60°/s. For both concentric and eccentric repetitions, subjects were exhorted to push/pull five continuous, reciprocal knee extensions and flexions as hard and fast as possible and to complete the full ROM.

### Criterion measures

For concentric and eccentric strength trials, we retained two sets of parameters for each individual contraction repetition of the knee: average peak moment (PM), which referred to the average peak moment of five trials and would be less affected by the artifact than the peak moment^33^; the moment of quadriceps and hamstring at specific 30°, 40°, 50°, 60°, 70°, 80° of flexion. And then H/Q ratios and 6 newly introduced indices of quadriceps and hamstring muscle co-contraction strength (the detailed mathematical forms are shown in Table 1) were calculated for the PM and 30°, 40°, 50°, 60°, 70°, 80° respectively.

**Table 1:** Definition of the original H/Q Ratios and two versions of newly introduced muscle strength indices.

Definition	Index Name	Mathematical Form	Expression
Ratios of eccentric quadriceps (Qe) to concentric quadriceps (Qc) muscle average peak moment	Qe/Qc	Original: A/B	*Qe*/*Qc*
	SAM-QeQc	New Ver. 1: 2A/(A + B)	2*Qe*/(*Qe* + *Qc*)
	SRMS-QeQc	New Ver. 2: $\sqrt{2}{\rm{A}}/\sqrt{{{\rm{A}}}^{2}+{{\rm{B}}}^{2}}$	$\sqrt{2}Qe/\sqrt{Q{e}^{2}+Q{c}^{2}}$
Ratios of concentric hamstring (Hc) to concentric quadriceps (Qc) muscle average peak moment	Hc/Qc	Original: A/B	*Hc*/*Qc*
	SAM-HcQc	New Ver. 1: 2A/(A + B)	2*Hc*/(*Hc* + *Qc*)
	SRMS-HcQc	New Ver. 2: $\sqrt{2}{\rm{A}}/\sqrt{{{\rm{A}}}^{2}+{{\rm{B}}}^{2}}$	$\sqrt{2}Hc/\sqrt{H{c}^{2}+Q{c}^{2}}$
Ratios of eccentric hamstring (He) to concentric quadriceps (Qc) muscle average peak moment	He/Qc	Original: A/B	*He*/*Qc*
	SAM-HeQc	New Ver. 1: 2A/(A + B)	2*He*/(*He* + *Qc*)
	SRMS-HeQc	New Ver. 2: $\sqrt{2}{\rm{A}}/\sqrt{{{\rm{A}}}^{2}+{{\rm{B}}}^{2}}$	$\sqrt{2}He/\sqrt{H{e}^{2}+Q{c}^{2}}$

H/Q, hamstring to quadriceps muscle strength ratio; SAM, single-to-arithmetic-mean form indices, the single muscle strength of hamstring or quadriceps to the arithmetic mean of two muscle strengths; SRMS, single-to-root-mean-square form indices, the single muscle strength of hamstring or quadriceps to the root mean square of two muscle strengths; Ver., newly introduced muscle indices version; A and B correspond to hamstring or quadriceps muscle strength.

### Data Processing

Gravity-correct measurement data from the dynamometer was slightly smoothed by fifth-order Savitzky-Golay filter with the frame size 51. This type of filter was chosen so that possible essential differences could be recorded, and at the same time the time-velocity curve could be smoothed for further use. All repetitions were used in subsequent analysis, for not all the participants performed the strongest peak moment value for the first repetition, and the majority of participants did not perform fatigue for the last one. Hamstring and quadriceps moment data of concentric and eccentric motion was respectively normalized by weight and height of each participant^5^, that is

1 $${\rm{Normalized}}\,{\rm{Moment}}=\frac{{\rm{Moment}}}{{\rm{Height}}\times {\rm{Weight}}}$$

and the normalized moment values (m/s^2^) versus knee extension-flexion angle (°) curves were then obtained from the smoothed experimental data. Several participants did not manage to move their limbs within the predefined ROM for fatigue in one or two repetitions, and these data would be moved out for further analysis. All the remained curves were normalized by the ROM of corresponding participants and projected to the predefined ROM (20°–90°) so that they can be compared with each other. The knee moments of each repetition were assumed to be linear with joint angle, and they were evenly distributed by 100 points using cubic spline interpolation. Only the data between 26°–84° was taken into consideration for average peak normalized moment (m/s^2^) per participant to neglect inertia effect.

### Statistical Analysis

Custom-written Matlab (MathWorks, Natick, MA) programs were used to perform statistical analyses. Nonparametric statistics were calculated for the average peak knee moments, ratios of hamstring strength between ACLD side and the healthy side (HHR)/ratios of quadriceps strength between ACLD side and the healthy side (QQR), H/Q ratios, the newly introduced SAM indices and the SRMS indices at 30°, 40°, 50°, 60°, 70°, 80° of knee flexion^34^. Firstly, the experimental data and computed ratios were checked for homogeneity of variance using Bartlett’s test. Then, a Friedman ANOVA by Ranks was chosen to assess whether there were significant differences in average peak knee moments, HHR/QQR ratios, H/Q ratios, SAM indices and SRMS indices^35^. It was also applied to detect differences among the values of the above variables by angle. When Friedman’s test was significant, the next step was to perform Wilcoxon signed Rank-test as post hoc analyses. The significance level was set as *P* < 0.05. Furthermore, Cohen’s d (*d*) was used as a measure of effect size.

## Results

### Average normalized moment-angle characteristics

In the case of concentric motion (Table 2), there was a significant difference in the mean normalized moment values between ACLD and the uninvolved limb for both extension and flexion motion. Post hoc analysis revealed that the extension normalized moment in ACLD knees decreased remarkably at all the angles (*P* < 0.010, QQR < 0.85). Normalized moment differences of knee flexion were smaller by angle (HHR < 0.85 for above 60°), and no significant differences were identified for 30° (*P* = 0.092, HHR = 0.92). In eccentric contraction mode (Table 2), no significant differences were identified for knee extension (*P* > 0.050), and the ACLD side was 13%-19% lower than the healthy side (QQR ≥ 0.85). There were statistically significant differences of normalized mean moment between the two sides at 80° knee flexion (*P* = 0.040), yet at other angles there was no significant difference (HHR ≥ 0.85, *P* ≥ 0.179) (see Supplementary Fig. S1).

**Table 2:** Mean normalized moment (m/s^2^) for knee flexion and extension (N = 46).

Knee	(°)	Concentric				Eccentric			
		Healthy (m/s^2^) Mean ± SD	ACLD (m/s^2^) Mean ± SD	Diff (%)	HHR/QQR Mean ± SD	Healthy (m/s^2^) Mean ± SD	ACLD (m/s^2^) Mean ± SD	Diff (%)	HHR/QQR Mean ± SD
Ext	PM	0.82 ± 0.26	0.57 ± 0.25	30.51**	0.80 ± 0.53^Δ^	0.64 ± 0.19	0.54 ± 0.20	14.77*	0.93 ± 0.48
	30	0.48 ± 0.18	0.31 ± 0.13	36.07**	0.77 ± 0.57^Δ^	0.56 ± 0.19	0.46 ± 0.20	17.27	0.91 ± 0.47
	40	0.63 ± 0.21	0.42 ± 0.18	33.66**	0.77 ± 0.51^Δ^	0.60 ± 0.19	0.51 ± 0.21	14.84	0.94 ± 0.51
	50	0.72 ± 0.23	0.49 ± 0.21	32.93**	0.77 ± 0.30^Δ^	0.59 ± 0.18	0.51 ± 0.21	13.51	0.94 ± 0.53
	60	0.79 ± 0.25	0.54 ± 0.24	30.83**	0.81 ± 0.55^Δ^	0.55 ± 0.18	0.46 ± 0.19	15.03	0.94 ± 0.56
	70	0.77 ± 0.26	0.55 ± 0.25	29.26**	0.84 ± 0.61^Δ^	0.48 ± 0.16	0.40 ± 0.17	15.80	0.94 ± 0.60
	80	0.65 ± 0.22	0.46 ± 0.21	28.59**	0.84 ± 0.59^Δ^	0.37 ± 0.15	0.30 ± 0.14	18.85	0.95 ± 0.69
Flex	80	0.42 ± 0.13	0.30 ± 0.13	28.84**	0.80 ± 0.46^Δ^	0.59 ± 0.22	0.48 ± 0.26	18.28*	0.90 ± 0.53
	70	0.48 ± 0.14	0.35 ± 0.14	26.47**	0.81 ± 0.42^Δ^	0.74 ± 0.26	0.64 ± 0.27	12.86	0.95 ± 0.42
	60	0.51 ± 0.15	0.38 ± 0.15	24.96**	0.83 ± 0.45^Δ^	0.79 ± 0.28	0.71 ± 0.29	10.13	1.00 ± 0.48
	50	0.53 ± 0.15	0.41 ± 0.15	22.37*	0.87 ± 0.48	0.73 ± 0.26	0.65 ± 0.24	11.50	0.99 ± 0.48
	40	0.52 ± 0.16	0.42 ± 0.15	19.81*	0.91 ± 0.52	0.59 ± 0.21	0.52 ± 0.20	11.45	1.00 ± 0.50
	30	0.49 ± 0.14	0.41 ± 0.15	16.88	0.92 ± 0.50	0.35 ± 0.16	0.30 ± 0.14	15.43	1.06 ± 0.79
	PM	0.55 ± 0.15	0.44 ± 0.15	19.98*	0.89 ± 0.47	0.84 ± 0.28	0.75 ± 0.28	10.84	0.99 ± 0.46

All the average moments were averaged by the height and weight of each corresponding participant. Single upper asterisk *denotes significant differences at *P* < 0.05, double upper asterisk **denotes significant differences at *P* < 0.01, and single triangle ^Δ^denotes HHR/QQR below 0.85. SD, standard deviation; Ext, knee extension; Flex, knee flexion; ACLD, anterior cruciate ligament deficient side; HHR, ratios of hamstring strength between ACLD side and the healthy side; QQR, ratios of quadriceps strength between ACLD side and the healthy side; Diff, the difference of the average moments between the ACLD side and the healthy side.

### H/Q ratio values

**Figure 2:**
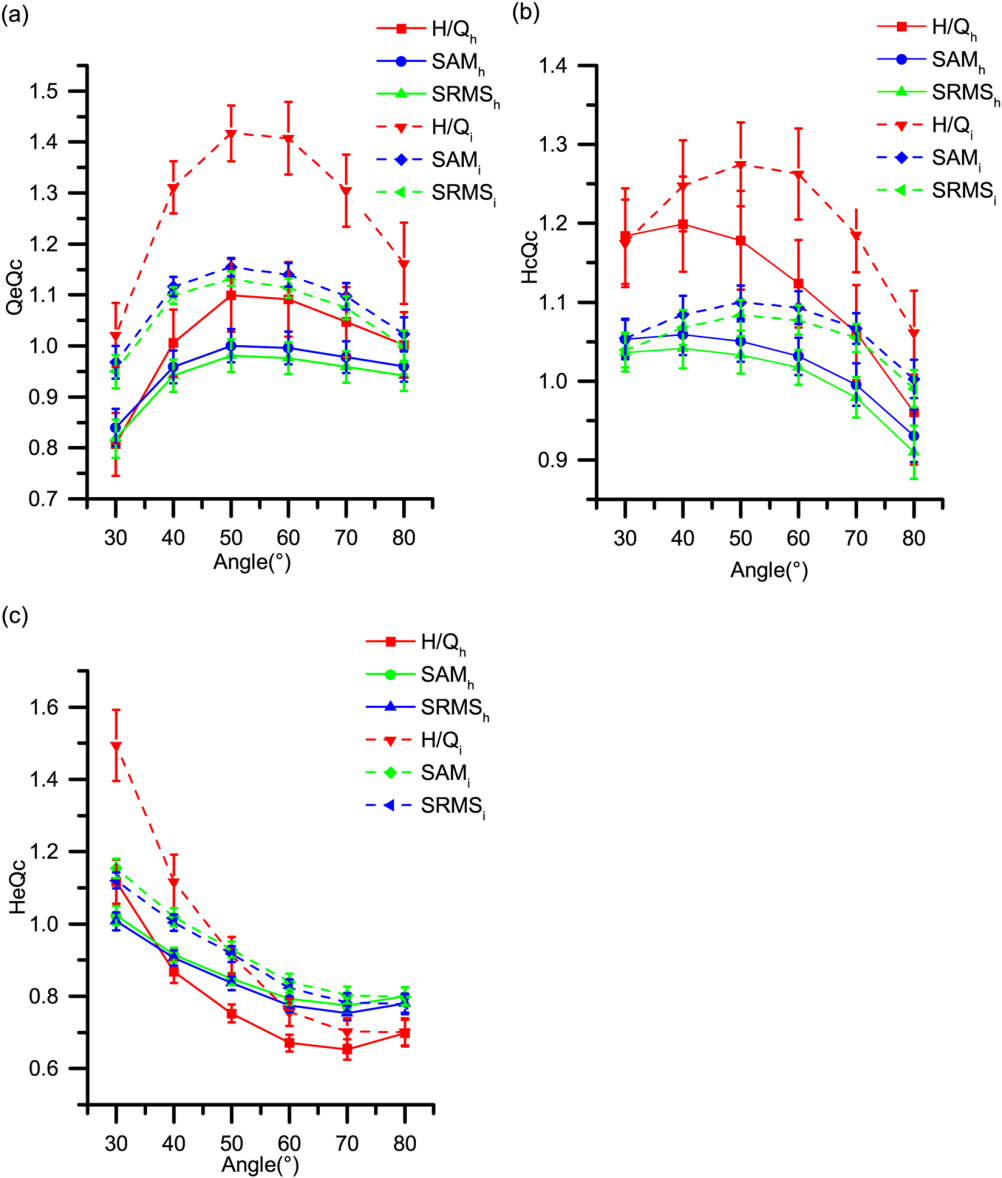
Mean values and standard error (s.e.m.) plot of H/Q ratio, SAM and SRMS indices. (**a**) The Qe/Qc, SAM-QeQc and SRMS-QeQc indices; (**b**) the Hc/Qc, SAM-HcQc and SRMS-HcQc indices; (**c**) the He/Qc, SAM-HeQc and SRMS-HeQc indices. SAM, single-to-arithmetic-mean form indices; SRMS, single-to-root-mean-square form indices; h, healthy limb (in solid lines); d, ACLD limb (in dashed lines).

The difference in Qe/Qc ratios between the healthy and ACLD knees was obviously discernible at 30°, 40°, 50° and 60° (*P* ≤ 0.032, Table 3), and the effect size at 40° (*d* = 0.766) and 50° (*d* = 0.739) was larger than the average peak moment Qe/Qc ratios (*d* = 0.654). A clear increase of the Hc/Qc ratios in the ACLD limbs (Table 3) can be observed in the average peak value (*P* = 0.032, *d* = 0.698), yet a fairly less remarkable change of the Hc/Qc ratios was observed at 30° and 40°knee angle (*P* ≤ 0.021, *d* ≥ 0.646), essentially consistent with the previously presented characteristics of H/Q ratios. The ACLD He/Qc deficit was significant at 30° and 40° (*P* ≤ 0.032, *d* ≥ 0.529), but the difference of the average peak moment He/Qc ratios was not significant (*P* = 0.080, Table 3). Regardless of the knee angle, one can observe high diversity of the H/Q ratio values (Fig. 2).

**Table 3:** H/Q ratios, SAM and SRMS form indices of peak moments and angle-specified moments (*N* = 46).

H/Q Ratios	Qe/Qc			Hc/Qc			He/Qc		
	Healthy Mean ± SD	ACLD Mean ± SD	Cohen’s *d*	Healthy Mean ± SD	ACLD Mean ± SD	Cohen’s *d*	Healthy Mean ± SD	ACLD Mean ± SD	Cohen’s *d*
PM	1.11 ± 0.48	1.41 ± 0.46	0.654*	0.69 ± 0.14	0.82 ± 0.23	0.698*	0.83 ± 0.31	1.05 ± 0.42	0.602
30°	0.81 ± 0.42	1.02 ± 0.43	0.506*	1.12 ± 0.41	1.49 ± 0.67	0.679*	1.31 ± 0.67	1.74 ± 0.94	0.529*
40°	1.01 ± 0.45	1.31 ± 0.35	0.766*	0.87 ± 0.22	1.12 ± 0.50	0.646*	1.03 ± 0.41	1.40 ± 0.76	0.600*
50°	1.10 ± 0.48	1.42 ± 0.37	0.739*	0.75 ± 0.17	0.91 ± 0.34	0.595	0.88 ± 0.33	1.17 ± 0.56	0.644
60°	1.09 ± 0.50	1.41 ± 0.48	0.643*	0.67 ± 0.16	0.76 ± 0.26	0.393	0.74 ± 0.27	0.96 ± 0.45	0.578
70°	1.05 ± 0.46	1.31 ± 0.48	0.548	0.65 ± 0.19	0.70 ± 0.25	0.225	0.68 ± 0.31	0.84 ± 0.40	0.437
80°	1.00 ± 0.44	1.16 ± 0.54	0.327	0.70 ± 0.25	0.70 ± 0.26	0.015	0.65 ± 0.37	0.73 ± 0.33	0.227
**SAM Ratios**	**SAM-QeQc**			**SAM-HcQc**			**SAM-HeQc**		
	**2Qe/(Qe + Qc)**			**2Hc/(Hc + Qc)**			**2He/(He + Qc)**		
PM	1.00 ± 0.21	1.14 ± 0.14	0.771*	0.81 ± 0.10	0.89 ± 0.12	0.706*	0.88 ± 0.19	0.99 ± 0.18	0.610
30°	0.84 ± 0.25	0.97 ± 0.22	0.548*	1.02 ± 0.18	1.15 ± 0.18	0.723*	1.07 ± 0.23	1.20 ± 0.23	0.561*
40°	0.96 ± 0.22	1.12 ± 0.13	0.887*	0.91 ± 0.13	1.02 ± 0.17	0.680*	0.97 ± 0.21	1.10 ± 0.23	0.591*
50°	1.00 ± 0.22	1.15 ± 0.12	0.863*	0.85 ± 0.12	0.93 ± 0.15	0.599	0.90 ± 0.19	1.03 ± 0.21	0.633*
60°	1.00 ± 0.22	1.14 ± 0.16	0.754*	0.79 ± 0.11	0.84 ± 0.15	0.360	0.83 ± 0.17	0.93 ± 0.21	0.558
70°	0.98 ± 0.21	1.10 ± 0.18	0.612*	0.78 ± 0.13	0.80 ± 0.16	0.192	0.78 ± 0.19	0.87 ± 0.20	0.472
80°	0.96 ± 0.20	1.02 ± 0.22	0.297	0.80 ± 0.16	0.80 ± 0.18	0.005	0.74 ± 0.22	0.80 ± 0.21	0.308
**SRMS Ratios**	**SRMS-QeQc**			**SRMS-HcQc**			**SRMS-HeQc**		
	$\sqrt{{\bf{2}}}{\bf{Qe}}/\sqrt{{\bf{Q}}{{\bf{e}}}^{{\bf{2}}}{\boldsymbol{+}}Q{{\bf{c}}}^{{\bf{2}}}}$			$\sqrt{{\bf{2}}}{\bf{Hc}}/\sqrt{{\bf{H}}{{\bf{c}}}^{{\bf{2}}}{\boldsymbol{+}}Q{{\bf{c}}}^{{\bf{2}}}}$			$\sqrt{{\bf{2}}}{\bf{He}}/\sqrt{{\bf{H}}{{\bf{e}}}^{{\bf{2}}}{\boldsymbol{+}}Q{{\bf{c}}}^{{\bf{2}}}}$		
PM	0.98 ± 0.21	1.12 ± 0.11	0.813*	0.79 ± 0.11	0.88 ± 0.13	0.702*	0.86 ± 0.20	0.98 ± 0.18	0.602
30°	0.82 ± 0.26	0.95 ± 0.22	0.551*	1.01 ± 0.17	1.12 ± 0.15	0.707*	1.04 ± 0.20	1.15 ± 0.18	0.552*
40°	0.94 ± 0.21	1.10 ± 0.11	0.918**	0.91 ± 0.14	1.00 ± 0.15	0.658*	0.96 ± 0.21	1.07 ± 0.20	0.566*
50°	0.98 ± 0.22	1.13 ± 0.10	0.892**	0.84 ± 0.13	0.92 ± 0.15	0.585	0.89 ± 0.21	1.01 ± 0.20	0.615*
60°	0.98 ± 0.21	1.11 ± 0.13	0.785*	0.77 ± 0.13	0.82 ± 0.15	0.344	0.81 ± 0.19	0.92 ± 0.21	0.543
70°	0.96 ± 0.21	1.07 ± 0.15	0.628	0.75 ± 0.14	0.78 ± 0.17	0.184	0.75 ± 0.21	0.85 ± 0.21	0.478
80°	0.94 ± 0.20	1.00 ± 0.21	0.279	0.78 ± 0.17	0.78 ± 0.19	0.004	0.71 ± 0.23	0.78 ± 0.22	0.331

Single upper asterisk *denotes significant differences at *P* < 0.05, and double upper asterisk **denotes significant differences at *P* < 0.01. SD, standard deviation; ACLD, anterior cruciate ligament deficient side; PM, the peak moment; Cohen’s *d*, effect size between the ACLD limb and the healthy limb. H/Q, hamstring to quadriceps ratios; SAM, single-to-arithmetic-mean form indices; SRMS, single-to-root-mean-square form indices; Qe, quadriceps eccentric contraction; Qc, quadriceps concentric contraction; He, hamstring eccentric contraction; Hc, hamstring concentric contraction.

### SAM/SRMS muscle strength indices

The generalized SAM/SRMS form QeQc indices showed marked increase in the ACLD limb at 30°–60° knee angle, extremely significant for SRMS-QeQc at 40° and 50°(*P* < 0.01, *d* ≥ 0.892). And the effect size of the corresponding average peak value ratios (*d* ≤ 0.813) was lower than that at 40° and 50° (*d* ≥ 0.867). As for SAM-HeQc and SRMS-HeQc ratio, significant differences were noticeable at 30°, 40° and 50°(*P* < 0.031, *d* ≥ 0.552), whereas no discernable decrease could be observed of the corresponding average peak moment ratios (*P* ≥ 0.053). Furthermore, the SAM-HcQc and SRMS-HcQc ratios presented a significant increase at ACLD side at 30° and 40° (*P* ≤ 0.029). And the effect size of the average peak moment ratio (*d* = 0.706) was still lower than the indices at the 30° of knee flexion (*d* = 0.723). Of note is that the variance of SAM and SRMS ratios was obviously decreased in the whole ROM (shown in Fig. 2), meaning a marked increase of robustness (Table 4).

**Table 4:** Statistic difference and effect size of the H/Q ratios, the SAM and SRMS indices by knee angle (*N* = 46).

Indices	PM	30°	40°	50°	60°	70°	80°
Qe/Qc	○+	○+	○+	○+	○+	×+	×−
SAM-QeQc	○+	○+	○++	○++	○+	○+	×−
SRMS-QeQc	○++	○+	○○++	○○++	○+	×+	×−
Hc/Qc	○+	○+	○+	×+	×−	×−	×−
SAM-HcQc	○+	○+	○+	×+	×−	×−	×−
SRMS-HcQc	○+	○+	○+	×+	×−	×−	×−
He/Qc	×+	○+	○+	×+	×+	×−	×−
SAM-HeQc	×+	○+	○+	○+	×+	×−	×−
SRMS-HeQc	×+	○+	○+	○+	×+	×−	×−

Single circle (○) denotes significant differences at *P* < 0.05, double circle (○○) denotes significant differences at *P* < 0.01, and single cross (×) denotes no statistical significant differences. Double transverse line (−) denotes very small effect size at Cohen’s *d* is less than 0.20, single transverse line (−) denotes small effect size at Cohen’s *d* from 0.20 to 0.50, single cross (+) denotes medium effect size at Cohen’s *d* from 0.50 to 0.80, an*d* double cross (++) denotes large effect size at Cohen’s *d* over 0.80. H/Q, hamstring to quadriceps muscle strength ratios; PM, peak moment; SAM, single-to-arithmetic-mean form indices; SRMS, single-to-root-mean-square form indices; Qe, quadriceps eccentric contraction; Qc, quadriceps concentric contraction; He, hamstring eccentric contraction; Hc, hamstring concentric contraction.

## Discussion

The objective of this study was to investigate the angle-specific moments, H/Q ratios and newly introduced muscle strength indices, which were important for knee dynamic function following unilateral ACL rupture^17^. Our hypotheses were basically supported by the results. At concentric mode, ACLD individuals revealed the largest quadriceps strength deficits for the injured limbs at less than 40° knee angle. Compared with related peak values, the Qe/Qc ratio change of ACL ruptured limbs was more obvious at 40° knee angle, while He/Qc ratios remained the same effect size. Moreover, the most evident diagnostic ratios among all the muscle co-contraction strength indices listed would be SRMS-QeQc ratios at 40° knee angle (*d* = 0.918) in statistical sense.

Contrary to common belief, the average quadriceps peak moment of concentric motion was almost the same as the eccentric average peak moment values, yet similar phenomenon had been reported as a special case in literature^36^. Comparing relative muscle strength values between the injured and the uninjured limb, the largest deficits of concentric quadriceps strength were established for less than 40° of knee flexion, in consistent with the literature^17^. The validity of the widespread use of the peak moment as the only measurement criterion was called into question by such an outcome. During isokinetic or isometric extension exercises, the peak ACL forces occurred at knee angles of around 35°^37^. And the peak anterior tibial shear force, referring to the maximal value of the resultant joint shear force in the anterior direction^38^, occurred at about 37° knee flexion during the jump landing task which is a common functional activity inherent to many sports and has been identified as a mechanism for non-contact ACL injury^39^. The contraction force of the quadriceps muscle during knee extension produced substantial anterior directed shear of the tibia relative to the femur at extended joint angles^40^. This shear can be counteracted not only by the ACL but also by hamstring coactivation^40^. Thus, low muscle strength of the hamstring relative to quadriceps has been proposed to increase the risk of noncontact knee joint injuries^12^, which was the original intention of introducing the H/Q ratio.

The H/Q ratio has been used as a guideline for managing strength training or injury rehabilitation by assessing changes in the ratios after training or injury^41^. The average peak moment Hc/Qc ratio and He/Qe ratio have been studied in ACLD subjects^15^. A greater difference between the peak moment Hc/Qc ratio of the injured and sound knee was associated with a less successful outcome of rehabilitation. A peak moment Hc/Qc ratio of 0.6 has been usually regarded as normative data^33^. But in our study, the average value of this ratio for the healthy limb was 0.69, partly because ACL injury could also affect its opposite side, resulting in bilateral quadriceps activation deficits after ACL injury^40^. Quadriceps contraction would probably increase tibial translation in the sagittal unstable knee, but hamstring contraction could reduce tibial translation^42^. An elevated H/Q ratio resulting from diminished quadriceps strength may indicate better H/Q strength balance. However, quadriceps weakness has been proven to be strongly associated with altered biomechanics in gait^43^ and may be linked to the pathogenesis of posttraumatic osteoarthritis^44^.

It has recently been suggested that the agonist-antagonist strength relationship for knee extension and flexion may be better described by a functional He/Qc or Hc/Qe ratio compared to conventional ratios (e.g. Hc/Qc or He/Qe)^12^. However, peak moment may give limited information about the muscle performance during the full selected range of motion^9^. While Qe peak moment generated for testing at 60°/s was attained at 73°, Hc peak moment was attained at 31°^38^. These findings illustrated the limitations of using peak moment to predict loading during functional activities^38^, although the peak moment He/Qc increase was fairly higher than angle-specific values. Therefore, our research focused on functional He/Qc ratios at 30°–50° of knee flexion which could be important for knee dynamic function. Functional He/Qc ratio deficit of the ACLD limb was only noticeable at 30° and 40°, yet the decrease in the peak moment He/Qc only showed marginally significant difference (*P* = 0.080), in consistence with the findings of Gibson *et al*.^45^. Moreover, Aagaard *et al*. only examined the He/Qc ratio below 50° of knee flexion, and hold the view that its value should be always above 1.0^12^, while our findings have shown that the He/Qc ratios for both the uninvolved and the ACLD limb were all below 1.0 at flexed knee joint position (≥60°). It was due to elongated force-length conditions for the quadriceps and shortened one for hamstring at flexed knee joint angles. These values may well reflect the real contraction force-length properties for the knee muscles, and may elucidate the change of the hamstring and quadriceps biomechanical profiles following ACL rupture.

Moreover, the Qe/Qc, Hc/Qc, and He/Qc indices of both average peak moment and the 30°, 40°, and 50° angle-specific moments were at least marginally significantly increased (*P* ≤ 0.080) on the ACLD side. These findings agree with numerous previous studies^7, 8, 17, 43, 46^ but disagree with others^45^. Differences in patient characteristics, especially different isokinetic dynamometers, confound direct comparisons among studies. For example, gravity correction was not used on the Kin-Com dynamometer, but in our study, gravity effect was corrected throughout the range of motion during all tests. Furthermore, there still have been debates about the menstrual cycle effects on the biomechanical profiles of women with ACL injury. Bell *et al*.^47^ highlighted gender difference on ACL reconstruction, yet Chaudhari *et al*.^48^ reported that hormone cycling in women did not affect knee joint biomechanics. Therefore, our study was confined to the use of male subjects to avoid any potential variability associated with gender effects.

**Figure 3:**
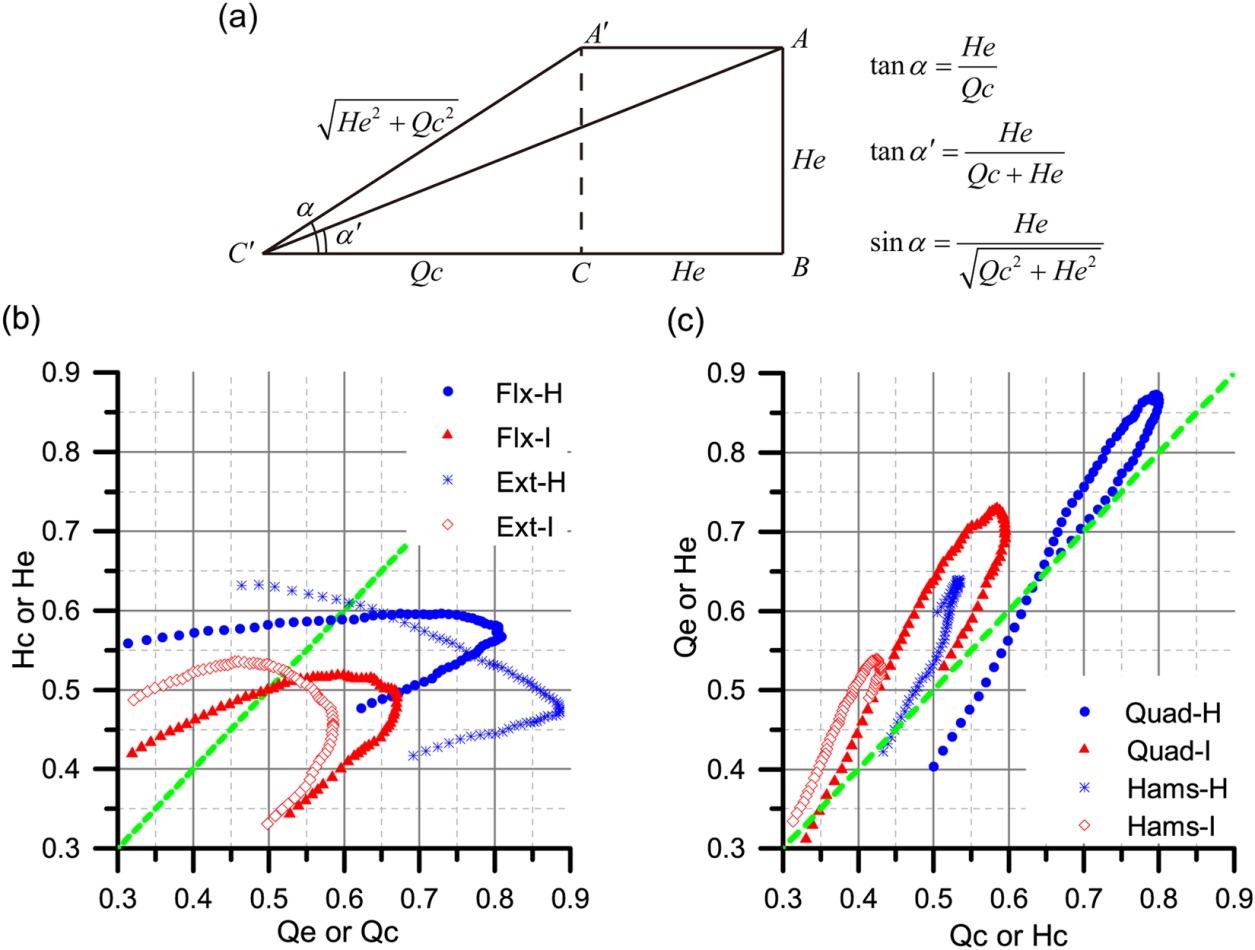
Relationship among different knee muscle strength indices. (**a**) Geometric description of the H/Q ratio and the SAM/SRMS ratios. He, hamstring eccentric contraction; Qc, quadriceps concentric contraction. (**b**) Illustration of knee flexion and extension motion. Flx, knee flexion; Ext, knee extension; H, healthy limb; I, ACLD limb; Qe, quadriceps eccentric contraction; Hc, hamstring concentric contraction. (**c**) Illustration of quadriceps and hamstring strength in concentric and eccentric modes. Quad, quadriceps; Hams, hamstring.

Another interesting finding of this study was two new versions of muscle co-contraction indices with less variance and more clinical accuracy, compared with the H/Q ratio. The H/Q ratio depended on many factors^35^, and the values of this ratio therefore had a fairly wide range (shown in Fig. 2), especially when some participants had severe muscle weakness in one of their limbs. Such numerical fluctuation could lead to clinical unreliability of the indices. Therefore, this led to our research of novel indices of hamstring and quadriceps co-activation. The geometric meanings of the novel indices were shown in Fig. 3. The H/Q ratio was a tangent function of the triangle ∠*A*′ *CC*′, which would become singularity when *α* approximates to 90°. At this time, the quadriceps strength tested by isokinetic concentric motion would come close to zero. In order to avoid similar circumstances, another tangent function, tan*α*′ of the triangle ∠*ABC*′, was introduced to replace the original one. Taking SAM-HeQc as an example, the denominator of the He/Qc ratio was replaced by the arithmetic mean of the hamstring and quadriceps strength, preventing possible singularity. Another method of eliminating singularity was to change the mathematical form of trigonometric function by using sinusoidal function. The SAM/SRMS form QeQc, HcQc, and HeQc indices of both average peak moment and the 30°, 40°, and 50° angle-specific moments were at least marginally significantly increased (*P* ≤ 0.10) on the ACLD side, consistent with the conventional one, and this procedure could decrease the ranges evidently, supporting such an approach.

In the computational biomechanics field, muscle recruitment of the musculo-skeletal system can be determined by an optimization problem, where the “polynomial criteria” was often used as the cost function^49^. The polynomial criteria can be interpreted as minimizing weighted average of muscle forces, and the mathematical form is

2 $$G({{\bf{f}}}^{(M)})=\sum _{i=1}^{{n}^{(M)}}{(\frac{{f}_{i}^{(M)}}{{N}_{i}})}^{p}$$

where *n* ^(*M*)^ is the number of muscles, ${f}_{i}^{(M)}$ are the muscle forces, *N*_*i*_ are the normalization factors, and *p* is the power exponent. This criterion has important implications for rehabilitation and surgical planning. Quadriceps and hamstring are the main agonist-antagonist muscle groups that coexist in knee extension motion. According to equation (2), the arithmetic mean (AM) and the root mean square (RMS) values of hamstring eccentric and quadriceps concentric muscle strength (He + Qc or He^2^ + Qc^2^) might be two acceptable muscle cost functions. They both predict synergistic muscle behavior, for the linear combination (*p* = 1) is a measurement of the limb endpoint stiffness^23, 50^, and the quadratic cost function (*p* = 2) tends to minimize endpoint positional errors^25^. After ACL injury, the constraints of knee mechanical system are diminished especially at extended knee joint motion^36^, resulting in the decrease of knee cost function and changed muscle co-activation strategies. Similarly, from our aspect of view, such a cost function can be extended to assess a single muscle group function. For example, quadriceps has two fundamental functions: concentric contraction for voluntary extension and eccentric contraction for flexion stability. According to the literature, ACL rupture could evoke neurophysiological abnormality in quadriceps, leading to its atrophy and activation failure^43^. Muscle strength deficit may lead to quadriceps strength rebalance between such two functions, similar to the muscle residual force enhancement phenomenon, which has been regarded as a combination between an active and a passive component^51, 52^. Therefore, the AM and RMS average of concentric and eccentric quadriceps strength (Qe + Qc or Qe^2^ + Qc^2^) could be a reasonable evaluating indicator for muscle biomechanical dysfunction. In fact, Qe/Qc ratio has already been proved a more valid index of muscular imbalance than Hc/Qc^53^. Compared with the H/Q ratio, SAM and SRMS formed ratios measured voluntary/passive muscle captivity in related single muscle (QeQc) or muscle group function (HeQc) cost, directly associated with the rehabilitation^16^.

The relationship among various angle-specific indices of quadriceps and hamstring strength was shown in Fig. 3. Figure 3(a) illustrated the knee muscle functional differences between healthy limbs and ACLD limbs, in which quadriceps strength was used as abscissa against hamstring strength as ordinate. As for knee extension, the slope of Qc-He curve He/Qc (tan*α*) was above 1 at below 40° knee angle for healthy limbs, yet the threshold knee angle value was up to 52° for ACLD limbs. The more even change of He/Qc ratios for ACLD limbs (Table 3) resulted in the relatively smoothness of the ACLD Qc-He curve (Fig. 3), similar to the phenomenon of ACL reconstructed subjects shown in literature^19^. The same analysis method was also applicable to knee flexion motion, but at this time Qe-Hc curves for both healthy and ACLD limbs were geometrically more similar. Figure 3(b) was an illustration of quadriceps and hamstring muscle strength abilities. In this case, muscle strength in concentric mode was used as abscissa, and eccentric strength as ordinate. Both Qc-Qe and Hc-He curves have shown a sharp turning point, almost coincident with the maximum Qe^2^ + Qc^2^ and He^2^ + Hc^2^ points. We found that the maximum Qe^2^ + Qc^2^ strength occurred at 63° knee angle for healthy limbs, albeit 62° for ACLD ones. However, the maximum He^2^ + Hc^2^ strength was found at 43° knee angle for both healthy and ACLD limbs. The turning points were in consistent with the optimal knee angles with maximum quadriceps and hamstring strength respectively (Table 3), which meant that this criteria was a good combination of isokinetic concentric and eccentric muscle strength. In a word, the protocol shown in Fig. 3(b,c) could be an auxiliary method of qualitatively measuring muscle dysfunction of ACLD patients.

To minimize the variance which could confound the results, our patients were male and recruited from the same department. However, several limitations should be considered when interpreting our results. First, the small sample size (*N* = 46) was acknowledged, and it was recognized that this may have impacted the large variability in the test results. Second, no control group of healthy subjects was included in this study. Even though the sound leg as control has been an established approach when evaluating deficits after ACL injury^34, 54^. Other studies suggested that neuromuscular dysfunction and quadriceps strength loss after ACL injury also affect the sound side^55^. Furthermore, ACL injury occurs at a higher rate in females than males due to neuromechanical characteristics between sexes. Including a sound control group of both sexes would therefore enhance our understanding of the isokinetic quadriceps strength profiles of the ACL injury.

## Conclusion

In conclusion, concentric angle-specific quadriceps muscle moments and the Qe/Qc ratios at 40° knee angle demonstrated more significant deficits at ACLD side than the conventional used peak moment values. In addition, the newly defined SAM and SRMS muscle co-contraction strength indices could evidently enhance statistical robustness than the ordinary H/Q ratios by eliminating numerical singularity. Furthermore, among all indices, SRMS QeQc ratios have shown the strongest differences between the healthy and ACLD limbs with the largest effect size. These results suggested that, in isokinetic 60 °/s mode, thigh muscle strength deficits and change of SRMS QeQc indices at 40° of knee flexion might be used in documenting muscle function of ACLD patients.

## Electronic supplementary material

Supplementary Info File
