# Supplementary material for: Isokinetic angle-specific moments and ratios characterizing hamstring and quadriceps strength in anterior cruciate ligament deficient knees
Source: Sci Rep. 2017 Aug 4;7:7269. doi: 10.1038/s41598-017-06601-5 (PMC5544756; doi:10.1038/s41598-017-06601-5)
Supplement: Supplementary file 1 — Supplementary Info File [file 41598_2017_6601_MOESM1_ESM.doc]

**Isokinetic angle-specific moments and ratios characterizing hamstring and quadriceps strength in anterior cruciate ligament deficient knees**

**Hongshi Huang**^1+^**, Jianqiao Guo**^2+^**，Jie Yang** ^1^**, Yanfang Jiang** ^1^**, Yuanyuan Yu** ^1^**, Steffen Müller**^3^, **Gexue Ren** ^2^***,Yingfang Ao**^1^*

1 Institute of Sports Medicine, Peking University Third Hospital, Beijing, 100191, China

2 School of Aerospace Engineering, Tsinghua University, Beijing, 100084, China

3 Departments of Sports Medicine and Orthopedics, University of Potsdam, Potsdam, 14469, Germany

+ These authors are co-first authors.

* These authors are co-corresponding authors (aoyingfang@163.com and rengx@mail.tsinghua.edu.cn)

**Supplementary Info File**

In Fig. S1(a)-(d), thin filaments mark the average moment for 3-5 repetitions of each participant, and the thick dashed lines interpret the average curve for all the participants. Each figure clearly shows a significant diversity of mean peak moments. Some of the characteristics had oscillatory patterns in the whole ROM, especially for eccentric motions ^1, 2^, while others displayed a smoothed concave profile. In the concentric motion, local extremity of moments originated from inertial effect were discernible before 26°and after 84°, while for the eccentric ones only slight differences could be found in the initial and the final phase of the movement.

**Figure S1. Normalized knee muscle strength characteristics.** Muscle moment normalized by the weight and height vs. knee angle characteristics for healthy limb muscle eccentric contraction (a) / concentric contraction (b), anterior cruciate ligament deficient (ACLD) limb muscle eccentric contraction (c) / concentric contraction (d) and total average moment plot (e). In (a) to (d), thin filaments colored from pink to green showed the average angle-specified moment by each group. M, muscle moment; H, height; W, weight; Eq, quadriceps eccentric contraction; Eh, hamstring eccentric contraction; Cq, quadriceps concentric contraction; Ch, hamstring concentric contraction.

**References**

1. Bryant AL, Clark RA, Pua YH. Morphology of hamstring torque-time curves following ACL injury and reconstruction: mechanisms and implications. *J Orthop Res*. 2011; 29:907-914.

2. Czaplicki A, Jarocka M, Walawski J. Isokinetic Identification of Knee Joint Torques before and after Anterior Cruciate Ligament Reconstruction. *PLoS One*. 2015; 10:e0144283.
